# Standardized *Punica Granatum* Pericarp Extract, Suppresses Tumor Proliferation and Angiogenesis in a Mouse Model of Melanoma: Possible Involvement of PPARα and PPARγ Pathways

**Published:** 2019

**Authors:** Sima Seifabadi, Golnaz Vaseghi, Mustafa Ghannadian, Shaghayegh Haghjooy Javanmard

**Affiliations:** a ***Applied Physiology Research Center, Cardiovascular Research Institute, Department of Physiology, Isfahan University of Medical Sciences, Isfahan, Iran. ***; b ***Cardiovascular Research Center, Cardiovascular Research Institute, Isfahan University of Medical Sciences, Isfahan, Iran . ***; c ***Department of Pharmacognosy, School of Pharmacy and Pharmaceutical Sciences, Isfahan University of Medical Sciences, Isfahan, Iran.***; **Pomegranate Pericarp, Tumor Growth, Vascular Endothelial Growth Factor, Tumor Proliferation, Angiogenesis**

## Abstract

Melanoma is a challenging disease to treat. *Punica granatum* L. has a potential anticancer effect. This study determined the antiproliferative and antiangiogenic potential of the extract from pomegranate pericarp (PPE) in melanoma. Melanoma cells (1 × 10^6^) were injected to C57BL6 mice subcutaneously. On 8^th^ day, mice were randomly divided into 9 groups. Group 1 was considered as control and received distilled water. Groups 2 to 5 received 50, 100, 200 or 400 mg/kg of standardized PPE, orally. Group 6 received 400 mg/kg PPE and PPAR-γ antagonist (T0070907, 5 mg/kg/day). Group 7 received 400 mg/kg PPE and PPAR-α antagonist (GW6471, 10 mg/kg/day). Groups 8 and 9 received PPAR antagonists alone. On the 16th day, mice were euthanized and the tumor samples were analyzed by immunohistochemistry staining for Ki-67 and CD31. Vascular endothelial growth factor (VEGF) plasma level was determined by ELISA. PPE at the doses of 50, 100, 200, and 400 mg/kg decreased tumor weight to 1.28, 1.03, 0.82, and 0.58 g, respectively, in comparison with 1.46 g in control group. Tumor volume reduced to 2.1, 1.7, 1.35 and 0.95 cm3 at the mentioned doses, in comparison with 2.4 cm^3^ in control group (P < 0.05 for all groups). VEGF, Ki-67 and CD31 were decreased dose dependently in the treatment groups (P < 0.05). PPARα and PPARγ antagonists significantly reduced the extract effects (P < 0.05). It was concluded that PPE may have a potential implication in melanoma treatment through activation of PPARα and PPARγ receptors.

## Introduction

Melanoma is a metastatic type of skin cancer with a poor prognosis. It is a rapid growing malignancy in both young and elderly people; current chemotherapies and radiotherapies are almost ineffective (1). In spite of recent advances in medicinal chemistry, the mortality rate of melanoma has not declined, representing the need for novel and more effective therapeutic approaches (2). 

Previous studies have shown that melanoma's growth and progression is highly dependent on angiogenesis; a physiological multistep process characterized by the formation of new capillaries from the preexisting vessels, essential for cancer growth (3, 4). Poor prognosis of melanoma is correlated to the promotion of angiogenesis and the overexpression of vascular endothelial growth factor (VEGF), as the most important inducer of angiogenesis (5). Plasma VEGF has a prognostic value in melanoma and the reduction of its expression inhibits both malignant melanoma cell proliferation and metastasis (6, 7, 8). 

Peroxisome proliferator-activated receptors (PPARs) including α, β, δ, and γ isoforms are ligand activated transcription factors and members of the nuclear hormone receptor superfamily (9). Previous studies have shown that PPARα and PPAR*γ* agonists suppress tumor growth in different cancer cells by angiogenesis inhibition (10, 11, 12, 13).

PPAR pathway is suggested to be a potential target in melanoma, and PPARα and PPAR*γ* receptors are overexpressed in melanoma cell lines compared to normal melanocytes (12, 13). Recently, the role of natural products and nutraceuticals in the cancer treatment has been highlighted (14). Pomegranate [*Punica granatum *L., (Punicaceae)] is a small tree, grown mainly in Iran, the United States, and India. It is a rich source of polyphenols, especially anthocyanins, tannins, flavonoids, and ellagic acid. Ellagic acid is known as the main component of pomegranate, responsible for the anticancer properties including the induction of cell cycle arrest, apoptosis, and the suppression of tumor formation (15, 16, 17). 

It is demonstrated that pomegranate fractions have preventive and curative effects in obesity, atherosclerosis, hyperlipidemia, inflammation, and bacterial infection (15). Moreover, pomegranate ellagitannin-rich fractions are determined to have antitumor properties (18, 19). Pomegranate peel extract is suggested to be more potent than other pomegranate preparations due to having a higher antioxidant activity and higher contents of phenolics, flavonoids, and proanthocyanidins (20). Pomegranate peel could inhibit premalignant colon cancer in animal models and demonstrate some promising effects on the invasion of prostate and breast cancers (21, 22, 23). However, there is little information available on the antitumor effects of *P. granutum *peel *in-vivo*. 

The present study determined the effect of PPE on melanoma tumor growth, plasma VEGF level, tumor proliferation (Ki-67 index), and angiogenesis (CD31 index). Furthermore, these effects are investigated to be through the PPARα and PPARγ pathways. 

## Experimental

*Plant material and preparation of the extract*

Pomegranate pericarp was purchased on November 2015, from the agricultural research center of Isfahan (Iran). It was identified by Mustafa Ghanadian, the professor of pharmacognosy, and a voucher specimen (no 2016) was deposited in the Department of Pharmaconosy, Isfahan University of Medical Sciences (Iran). Pomegranate pericarp was shade dried and macerated with ethanol containing 2% acetic acid at room temperature for 72 h. The filtered extract was concentrated in a rotary evaporator at 40 °C to a gummy extract. It was dried to a fine powder in a freeze drier and then stored at -20 °C until use (24).

*HPTLC Standardization of the Punica granatum extract*

 An accurate and repeatable high-performance thin-layer chromatography (HPTLC) method with the help of TLC scanner was done on the fruit pericarp extract for the quantification of the ellagic acid. Briefly, 20 g of the dried plant material was weighted and extracted with 100 mL ethanol: acetic acid (99:1), three times. The extracts were mixed and evaporated to dryness. The freeze dried extract (40 mg), containing the ellagic acid was solubilized in 1 mL of methanol. The sample was spotted in the form of 1 μL spots width on prewashed silica gel TLC AL foil 60 (20 cm × 10 cm with 0.2 mm thickness; E. Merck, Darmstad, Germany) using a Camag nanomat (CAMAG, Muttenz, Switzerland). A constant application rate of 150 nL/s was employed. The slit dimension was kept at 4 mm × 0.1 mm, and 20 mm/s scanning speed was employed. These parameters were kept constant throughout the analysis of the samples. The mobile phase consisted of toluene: ethyl acetate: formic acid: methanol (30-30-8-2) (25). Plates were developed in ascending order with a CAMAG twin trough glass tank which was pre-saturated with the mobile phase for 15 min; the length of each run was 8 cm. The determination was done at 280 nm using a TLC Scanner 3 (CAMAG, Muttenz, Switzerland). A standard calibration curve in the range of 100 - 800 µg/mL for quantitative analysis was prepared using different concentrations of ellagic acid (Sigma Aldrich, USA) as standard material (100, 200, 400, and 800 µg/mL). The relationship between the concentration and peak-height was measured using the minimum square method (R2 value).

*Cell culture*

B16-F10 melanoma cells, purchased from the National Cell bank of Iran (NCBI), were cultured in DMEM (supplemented with 4 mM -glutamine,4.5 g/L glucose, 10% FBS, 100 μg/mL streptomycin, 100 μg/mL penicillin) under 37 °C humidified air containing 5% CO_2_. When the cell monolayers were 80% confluent, they were washed with PBS and detached (with PBS containing 0.25% trypsin and 0.03% EDTA), then centrifuged. After removing the supernatant, cell pellets were suspended in PBS and counted.

*Animal*

Male C57BL6 mice, 6-8 week-old and 20-30 g weight were purchased from the Pasteur Institute of Iran. They had free access to water and standard pelleted chew. All protocols were designed and performed in accordance with the NIH Guidelines for the Care and Use of Laboratory Animals.

After 1 week of acclimatization, 1 × 10^6^ melanoma cells were injected subcutaneously (SC) to the right flank of each mouse with an insulin syringe (day 0). Primary tumors were developed on the 7^th^ day in all mice. On the day 8, the mice were randomly divided into nine groups of eight mice. Group 1 was kept as a control and received normal saline. Groups 2 to 5 received PPE orally once daily (50, 100, 200 or 400 mg/kg, respectively). In order to evaluate the mechanism of action of PPE, group 6 received GW6471 (PPAR α antagonist) 10 mg/kg/day, dissolved in dimethyl sulfoxide (DMSO), along with 400 mg/kg PPE. Group 7 received T0070907 (PPAR γ antagonist) intraperitoneally (5 mg/kg/day) dissolved in DMSO along with 400 mg/kg PPE. In order to ensure that PPAR antagonists do not have any specific effects on tumor growth, groups 8 and 9 received GW6471 (10 mg/kg/day) and T0070907 (5 mg/kg/day), respectively (26, 27).

At the day 16, animals were euthanized by pentobarbital. They were bled through the heart puncture and their plasma samples were separated. Furthermore, tumors were dissected, weighed and fixed in 10% buffered formalin. Tumor sizes were also measured as a prolate spheroid: V = (4/3×π× (a) 2× (b), where “a” is the half of the minor axis and “b” is the half of the major axis.

*Ki-67 evaluation*

The paraffin-embedded samples were cut at 5 µm thickness, dewaxed with xylene and then dehydrated through the graded alcohol. The samples were boiled for 10 min in a microwave oven in 10 mmol/L sodium citrate buffer for the antigen retrieval at pH 6.0. Mouse monoclonal antibody Ki67 (RTU-MMI, Novocastra - Germany) was applied to the sections (diluted to a concentration of 1:60) and then incubated for 30 min at room temperature. This antibody was detected by using a proprietary horse radish peroxidase enzyme labeled polymer (DAKO Envision - HRP) conjugated to the mouse secondary antibody. After the staining was developed with DAB, sections were counterstained with hematoxylin. The primary antibody was omitted in the negative controls. The number of mitosis was counted in five high-power fields (x 400) in the areas of the highest mitotic activity. Randomly selected cases were evaluated by two observers in order to control for inter-observer variation.

**Figure 1:**
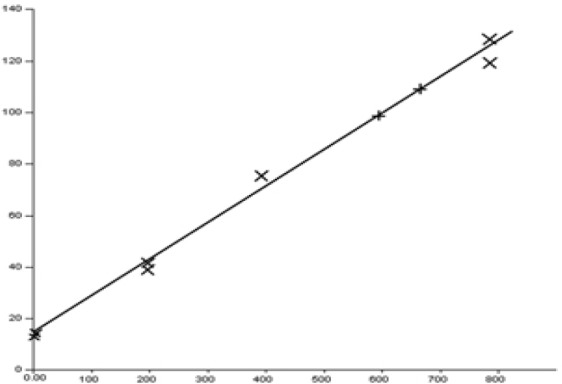
**Calibration curve of** ***Punica granatum*** **peel extract using HPTLC method. Using Camag TLC scanner and wincats software, the calibration curve was determined by the linear regression in the range of 100-800 µg/ml. The regression equation was y =0.1418 x +14.571, where X is the concentration of ellagic acid in sample (µg/mL) with the correlation co-factor (R2) of 0.9958**

**Figure 2.**
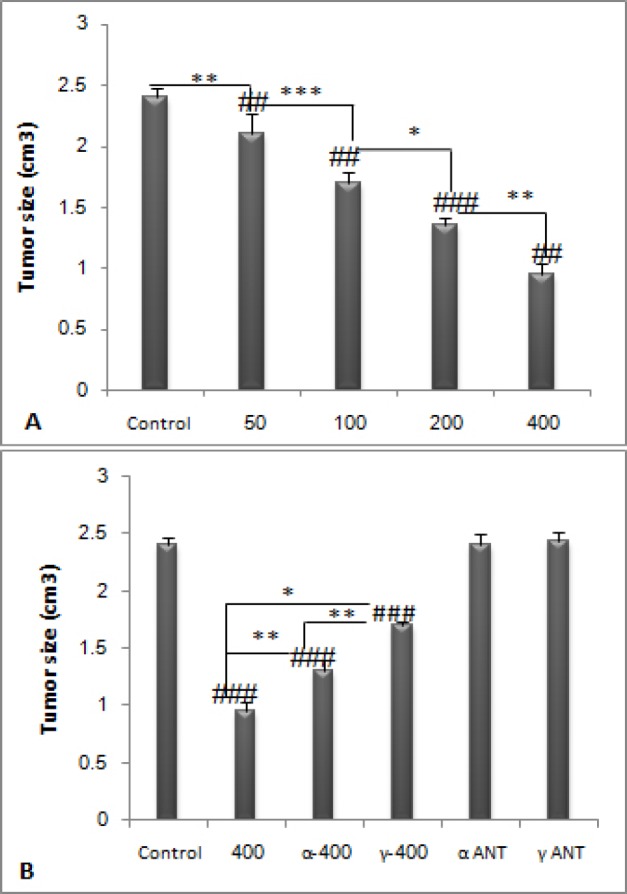
**Tumor sizes were measured on 16** **th** **day. PPE dose dependently decreased tumor size (** ***P*** **< 0.05). Administration of PPAR antagonists along with merely 400 mg/kg dose of PPE, led to the increase of tumor size comparing to 400 mg/kg dose (** ***P*** **< 0.05).** **#** ***P*** **< 0.05,** **##** ***P*** **< 0.01 and** **###** **P < 0.001 demonstrate significant values versus control,** * ***P*** **< 0.05** ** ***P*** **< 0.01 and** *** ***P*** **< 0.001 demonstrate significant values between groups. Data are shown as Mean ± S.D**

**Figure 3:**
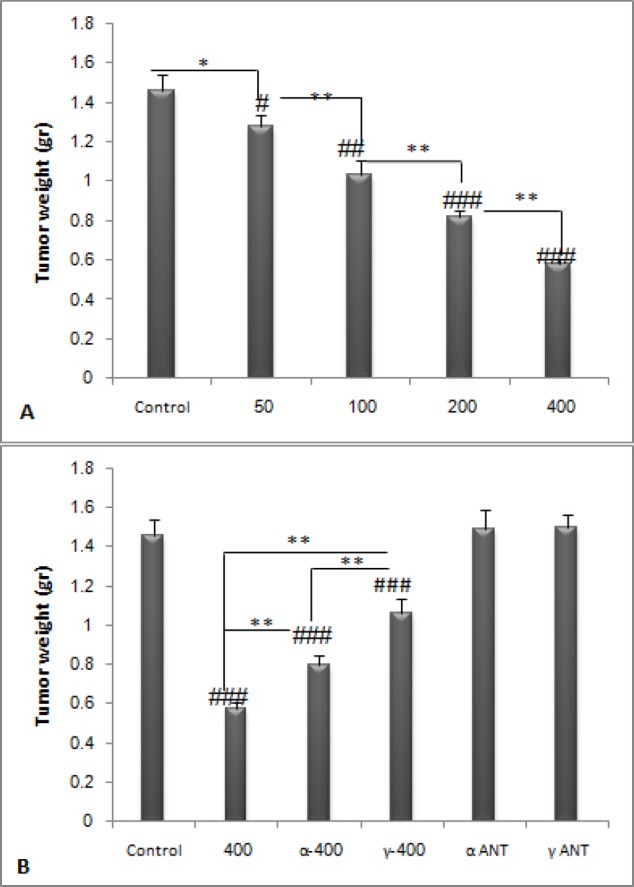
**Tumor weights on 16** **th** **day. PPE dose dependently decreased tumor weight (** ***P*** **< 0.05). Tumor weights of the two groups receiving PPAR antagonists along with only 400 mg/ kg dose of PPE, were significantly increased compared to 400 mg/kg dose (** ***P*** **< 0.01).** **#** ***P*** **< 0.05,** **##** ***P*** **< 0.01 and** **###** ***P*** **< 0.001 demonstrate significant values versus control,** * ***P*** **< 0.05,** ** ***P*** **< 0.01 and** *** ***P*** **< 0.001 demonstrate significant values between groups. Data are shown as Mean ± S.D**

**Figure 4:**
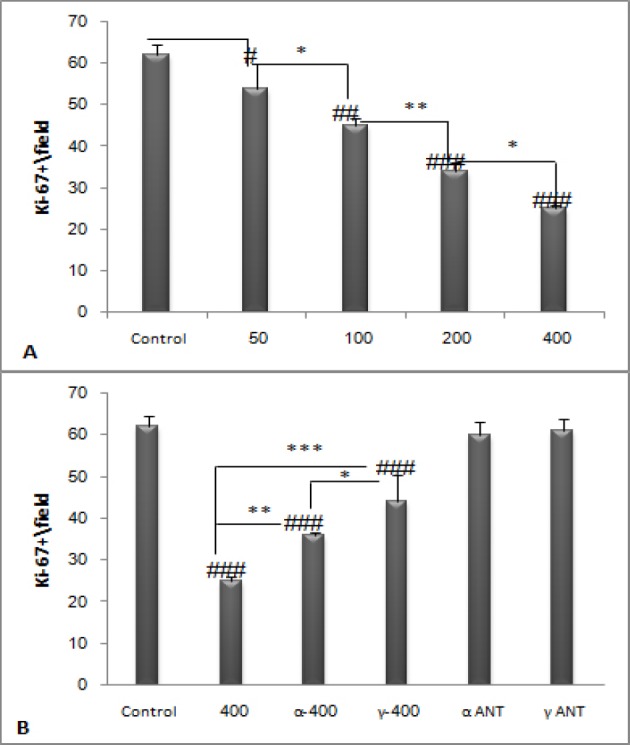
**Tumor sections were stained using immunohistochemistry for proliferative marker Ki-67. PPE dose dependently decreased Ki67 positive cells in tumor bearing mice (** ***P*** **< 0.05). Simultaneous administration of 400 mg/kg dose of PPE and PPAR antagonists increased Ki67 positive cells than merely 400 mg/kg dose of PPE (** ***P*** **< 0.05). #** ***P*** **< 0.05, ##** ***P*** **< 0.01 and ###** ***P*** < 0.001 demonstrate significant values versus control, * ***P*** < 0.05, ** ***P*** < 0.01 and *** ***P*** **< 0.001 demonstrate significant values between groups. Data are shown as Mean ± S.D**

**Figure 5:**
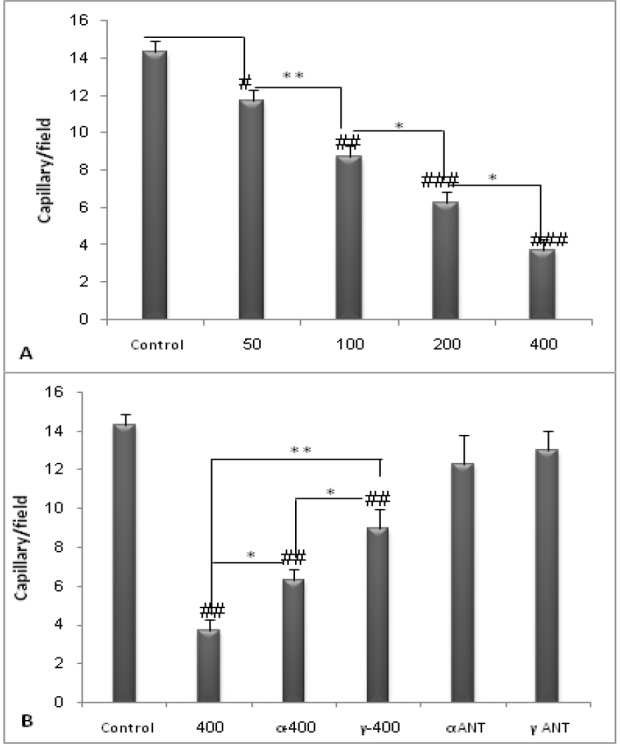
**The vascular density was measured in CD31-labeled vessels. PPE decreased capillary density dose dependently (** ***P*** **< 0.05). Coadministration of PPAR antagonist and PPE increased CD31 staining in comparison with the extract alone (** ***P*** **< 0.05).** **#** ***P*** **< 0.05,** **##** ***P*** **< 0.01 and** **###** ***P*** **< 0.001 demonstrate significant values versus control,** * ***P*** **< 0.05,** ** ***P*** **< 0.01 and** *** ***P*** **< 0.001 demonstrate significant values between groups. Data are shown as Mean ± S.D**

**Figure 6:**
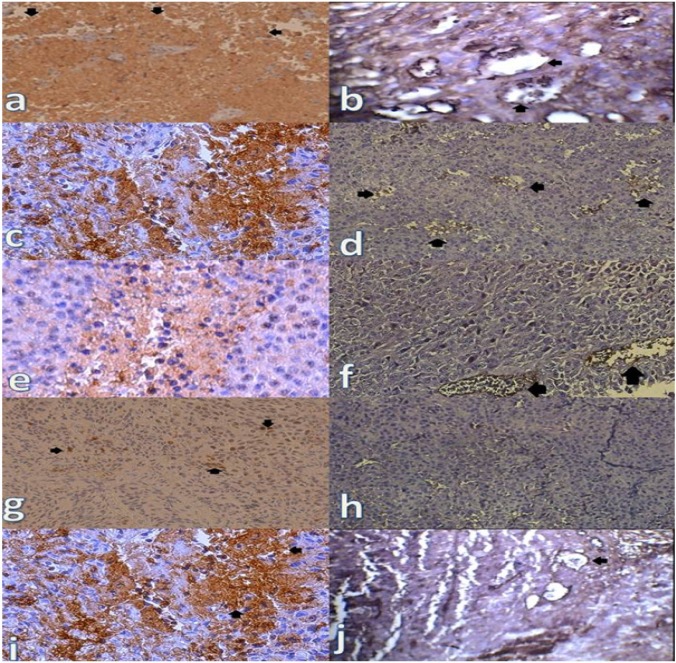
**Immunostaining of CD31 & Ki-67 in melanoma tumor, (a and b) Control, (c and d) 100mg/kg, (e and f) 200mg/kg, (g and h) 400mg/kg, (i and j) 400-γ. PPE dose dependently decreased CD31 & Ki-67 markers in the tumors (** ***p*** **< 0.05). Original magnification×400. Note the strong brown staining in tumors of control group compared to different doses of PPE. Also note the increase of the two markers in 400-γ group in comparison with merely 400 mg/kg dose of the extract**

**Figure 7:**
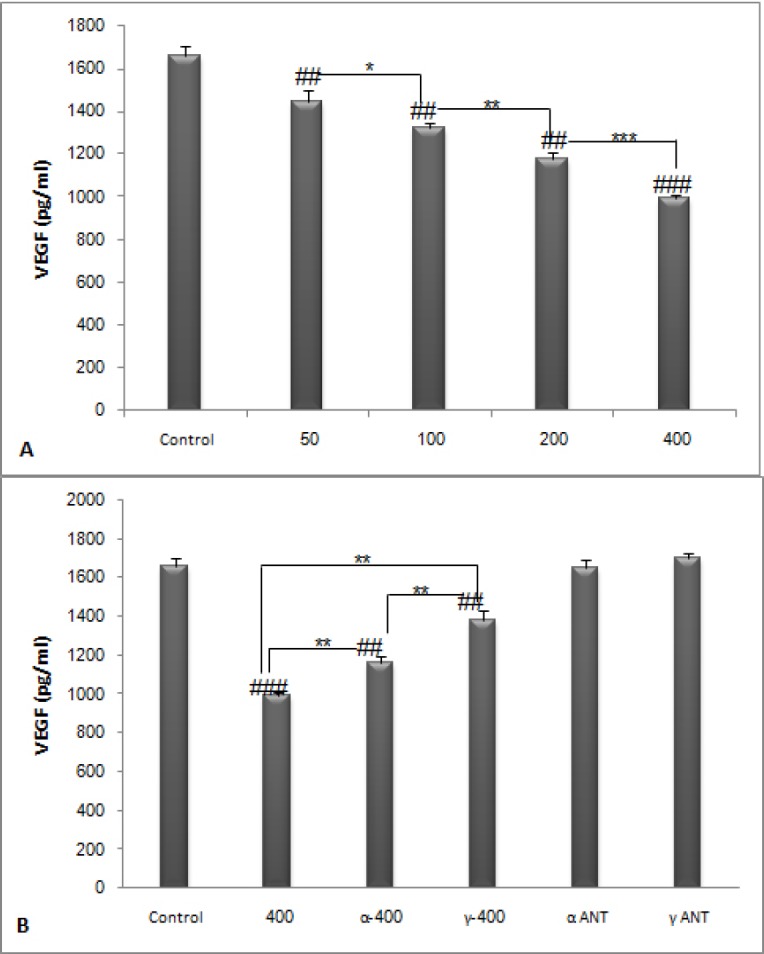
**The plasma concentration of VEGF. PPE dose dependently decreased plasma level of VEGF (** ***P*** **< 0. 05) and administration of PPAR antagonist and PPE increased plasma level of VEGF compared to the extract alone (** ***P*** **< 0. 01).** ***#*** ***P*** **< 0.05,** **##** ***P*** **< 0.01 and** **###** ***P*** **<0.001 demonstrate significant values versus control,** * ***P*** **< 0.05,** ** ***P*** **< 0.01 and** *** ***P*** **< 0.001 demonstrate significant values between groups**

**Data are shown as Mean ± S.D.**

*Capillary density assessment*

For endothelial cell immunostaining, the tumor samples were fixed in 10% neutral buffered formalin overnight, embedded in paraffin and cut in 5 µm. Then, the sections were deparaffinized in xylol followed by the rehydration in graded alcohol series. Endogenous peroxidase activity was blocked with 3% H_2_O_2_ in methanol. Antigen retrieval was performed on these sections by microwave irradiation for 15 min in citrate buffer 10 mM, pH 6.0. Sections were allowed to cool for 20 min and then washed in PBS. The slides were then incubated for 60 min with monoclonal antibodies (dilution 1/100) directed against mouse CD31 (ebioscience). The bound antibody was detected by the horseradish peroxidase enzyme labeled polymer conjugated to the mouse secondary antibody and visualized by diaminobenzidine as chromogen, then counterstained with hematoxylin. The paraffin embedded sections of normal samples were included as positive control. Negative control had the primary antibody replaced by phosphate buffered saline. The capillary density was measured at 400× in three separate fields from each tissue preparation; final vessel count was expressed as the mean number of vessels in these three fields.

*Determination of VEGF in plasma*

Plasma was isolated from the whole blood and the amount of VEGF in the plasma samples was measured using the enzyme-linked immunosorbent assay via reagents and recombinant standards (R&D Systems, Minneapolis, USA) according to the manufacturer’s instructions. The VEGF assay had a minimum sensitivity of 3.0 pg/mL.

*Statistical analysis*

The data were analyzed by the SPSS Statistics version 20 and expressed as the mean plus or minus the standard deviation (mean ± SD). The data were analyzed using an analysis of variance test (ANOVA), followed by a *post hoc* multiple comparison. *P* < 0.05 considered as a statistical significance.

## Results

*HPLC standardization of the P.granatum hydroalcoholic pericarp extract*

 The Rf value for ellagic acid was found to be 0.35 ± 0.02. By the aid of the Camag TLC scanner and Wincats software, the calibration curve was determined by linear regression in the range of 100-800 µg/mL. The regression equation was y = 0.1418 x + 14.571, where X was the concentration of ellagic acid in sample (µg/mL) with the correlation co-factor R² = 0.9958 (Figure 1). Then sample obtained from *P. granatum* peel extract was applied and its ellagic acid concentration was determined through the standard calibration curve. It was standardized to contain 1.57 ± 0.13 g ellagic acid per 100 g of the dried extract of *P. granatum* pericarp.

*Ki-67 evaluation*

We examined the proliferation in the mouse melanoma by using immunohistochemistry method for the proliferative marker Ki-67. PPE dose dependently reduced Ki-67 positive cells in tumor bearing mice (*P* < 0.05). Inhibition of PPAR receptors increased Ki-67 index compared to PPE alone (*P* < 0.01). Moreover, PPAR antagonists did not significantly affect the proliferative marker (Figure 4).

*Capillary density assessment*

Increasing dose of the extract significantly decreased mean number of vessels in tumor bearing mice (*P* < 0.05). Inhibition of PPAR receptors increased the capillary density compared to PPE (400 mg/kg) (*P* < 0.05). Moreover, PPAR antagonists did not significantly alter the capillary density. Samples of immunohistochemical staining in some of the experimental groups are presented in Figure 5 and 6.

*Determination of VEGF in plasma*

PPE dose dependently decreased the concentration of VEGF in plasma (*P* < 0.05). Inhibition of PPAR receptors increased the capillary density compared to PPE alone (*P* < 0.01) (Figure 7).

## Discussion

The present study showed that PPE dose dependently decreased tumor growth, proliferation, angiogenesis, and plasma VEGF. It is also observed that the inactivation of PPAR-α and PPAR-γ receptors declined the suppressive effect of PPE on melanoma tumor which indicates that PPE exerts its suppressive effects at least in part through activation of PPAR-α and PPAR-γ receptors. Furthermore, PPAR-γ inactivation more severely decreased the extract effects than PPAR-α inactivation demonstrating that the suppressive effect of the extract through PPAR-γ is more potential than PPAR-α.

Pomegranate fractions are demonstrated to have antiangiogenic and antiproliferative effects on human umbilical vein endothelial cells (HUVECs) (19). In a study by Sartippour *et al*., polyphenol rich extract of pomegranate (known as ellagitannins), from the pressed whole pomegranate, including skin and arils of the fruit, inhibited the proliferation of human prostate cancer cells and HUVECs. It also decreased prostate cancer xenograft size, tumor angiogenesis, and VEGF level (18). Furthermore, it was demonstrated that pomegranate pericarp polyphenols inhibited the proliferation of prostate cancer cells, induced cell apoptosis by induction of G_2_M arrest and loss of mitochondrial membrane integrity, inhibited cancer cell invasion through a Matrigel membrane, and suppressed tumor growth *in-vivo* (21). 

Previous studies have shown that PPARα ligands could suppress the growth of several cancer lines, including colon, endometrial and breast *in-vivo* and *in-vitro*. PPARα agonists including fenofibrate, suppress tumor growth through angiogenesis inhibition and it is demonstrated that the antiangiogenic activity of PPARα ligands is specifically dependent on the activation of this pathway (10, 11). PPAR*γ *is expressed in a variety of cancers, and PPAR*γ *ligands exert their antiproliferative effects on these cancer cells (12, 13). PPAR*γ *ligands are capable of inhibiting the angiogenesis, dominantly by targeting proliferation and migration of endothelial cells and inhibition of cancer malignant transformation. Therefore, targeting PPAR-*γ *pathway is introduced as a potential strategy in combination with conventional treatments (28). 

To the best of our knowledge, there have only been a few studies on PPAR pathway activation of pomegranate fractions (29, 30, 31). Pomegranate flower is introduced as a dual PPAR α/γ agonist and is suggested to have an implication in the treatment of diabetes (29). Punicic acid, as a conjugated linolenic acid isomer, ameliorates glucose homeostasis and obesity inflammation through the activation of PPARα and PPAR-γ (30). Antioxidant effects of pomegranate juice is maintained by the up-regulation of paraoxonase 2 (PON2) expressions, as a protector against the oxidative stress via PPARγ activation pathway (31).

It has been previously mentioned that, PPE mechanism of action is through both PPAR-γ and PPARα receptors and its effect through PPAR-γ is more potential. Some evidences about the role of PPAR-γ in melanoma progression explain this observation. Several studies have shown that PPAR-γ activation suppresses melanoma progression and metastasis. Considering the chemoresistance of current FDA-approved treatments of malignant melanoma, specific targeting of PPARγ is suggested to be a critical therapeutic approach (13). Since the activation of PPARα did not show antiproliferative effects on melanoma cells, this receptor was not suggested to be a therapeutic target in melanoma treatment (11, 12). 

However, here in this study we showed that pomegranate phytochemicals are good inhibitors of melanoma progression through both of the PPAR receptors.By the revolution of the therapeutic approaches of melanoma, from limited systemic options to the targeted therapies introducing new treatment approaches, PPAR receptors are one of those newly discovered challenging targets in the treatment of melanoma (32). This study showed that PPE is a mechanism-based chemopreventative agent that targeted one of the important molecular agents in melanoma.

However, it is not clear which component within PPE is exactly responsible for the observed effect. In a study the individual polyphenolic ingredients including ellagic acid, punicalagin, and tannins have shown synergistic effect on the antiproliferative, antiapoptotic and antioxidant activity; however, pomegranate juice showed a superior activity than purified ingredients (33). In another study by Lansky *et al*. dissimilar biochemical components from juice, peel and seed oil of pomegranate had a synergistic effect on proliferation and metastasis of human prostate cancer cells (34).

## Conclusion

In conclusion, PPE dose dependently decreased tumor size, weight, proliferation, and angiogenic markers. These effects are at least in part through PPARα and mostly PPAR-γ. Taken together, our observations suggest that PPE may have a potential implication in melanoma cancer treatment and is a good candidate for conducting future clinical trials in melanoma.
